# Antibiotic susceptibility profiles of *Mycoplasma synoviae* strains originating from Central and Eastern Europe

**DOI:** 10.1186/s12917-017-1266-2

**Published:** 2017-11-17

**Authors:** Zsuzsa Kreizinger, Dénes Grózner, Kinga M. Sulyok, Kristin Nilsson, Veronika Hrivnák, Dušan Benčina, Miklós Gyuranecz

**Affiliations:** 1 Institute for Veterinary Medical Research, Centre for Agricultural Research, Hungarian Academy of Sciences, Hungária körút 21, Budapest, 1143 Hungary; 2 Biotechnical Faculty, University of Ljubljana, Groblje 3, SI-1230 Domžale, Slovenia

**Keywords:** Antibiotic resistance, Chicken, Turkey, MIC, Microbroth dilution, *Mycoplasma synoviae*

## Abstract

**Background:**

*Mycoplasma synoviae* causes infectious synovitis and respiratory diseases in chickens and turkeys and may lead to egg shell apex abnormalities in chickens; hence possesses high economic impact on the poultry industry. Control of the disease consists of eradication, vaccination or medication. The aim of the present study was to determine the in vitro susceptibility to 14 different antibiotics and an antibiotic combination of *M*. *synoviae* strains originating from Hungary and other countries of Central and Eastern Europe.

**Results:**

Minimal inhibitory concentration (MIC) values of a total of 41 *M*. *synoviae* strains were determined by the microbroth dilution method. The strains were collected between 2002 and 2016 and originated from Hungary (*n* = 26), Austria (*n* = 3), the Czech Republic (*n* = 3), Slovenia (*n* = 3), Ukraine (*n* = 3), Russia (*n* = 2) and Serbia (*n* = 1). Tetracyclines (with MIC_50_ values of 0.078 μg/ml, ≤0.25 μg/ml and 0.5 μg/ml for doxycycline, oxytetracycline and chlortetracycline, respectively), macrolides (with MIC_50_ values of ≤0.25 μg/ml for tylvalosin, tylosin and tilmicosin), pleuromutilins (with MIC_50_ values of 0.078 μg/ml and ≤0.039 μg/ml for tiamulin and valnemulin) and the combination of lincomycin and spectinomycin (MIC_50_ 1 μg/ml (0.333/0.667 μg/ml)) were found to be the most effective antibiotic agents against *M*. *synoviae* in vitro. High MIC values were detected in numerous strains for fluoroquinolones (with MIC_50_ values of 1.25 μg/ml and 2.5 μg/ml for enrofloxacin and difloxacin), neomycin (MIC_50_ 32 μg/ml), spectinomycin (MIC_50_ 2 μg/ml), lincomycin (MIC_50_ 0.5 μg/ml) and florfenicol (MIC_50_ 4 μg/ml). Nevertheless, strains with elevated MIC values were detected for most of the applied antibiotics.

**Conclusions:**

In the medical control of *M. synoviae* infections the preliminary in vitro antibiotic susceptibility testing and the careful evaluation of the data are crucial. Based on the in vitro examinations doxycycline, oxytetracycline, tylvalosin, tylosin and pleuromutilins could be recommended for the therapy of *M*. *synoviae* infections in the region.

**Electronic supplementary material:**

The online version of this article (10.1186/s12917-017-1266-2) contains supplementary material, which is available to authorized users.

## Background

*Mycoplasma synoviae* is a cell wall-less pathogen which has significant economical impact in the poultry industry [1]. Clinical signs caused by this bacterium comprise synovitis and respiratory diseases in chickens and turkeys, and mainly in commercial egg layers the reduction of egg production and hatchability, and egg shell apex abnormalities [2]. The severity of the clinical signs may vary from sub-clinical to severe forms and is aggravated by the presence of other pathogens (e.g. infectious bronchitis virus, Newcastle disease virus, influenza A virus, *Escherichia coli* or other mycoplasmas) and inadequate housing conditions [3, 4].

The three main approaches for the control of the disease are eradication followed by prevention, vaccination or medication. While eradication and vaccination provide long-term solution for the control of mycoplasmosis, medication can be a prompt and effective tool to reduce the economic losses by mitigating egg transmission and clinical signs [1]. However, antibiotic susceptibility profile should first be determined to maximize treatment efficacy [5].

Due to their cell wall-less characteristic mycoplasmas are readily resistant to ß-lactam antibiotics and as they do not synthesize folic acid sulphonamides, sulfones or trimethoprim are ineffective against these pathogens also [6]. Natural resistance to erythromycin and other 14-membered ring macrolides was described in *M. synoviae* [7]. Mycoplasmas showed susceptibility to tetracyclines, fluoroquinolones and macrolides both in vitro and in vivo, and the efficacy of tiamulin and the combination of lincomycin and spectinomycin against *M. synoviae* under experimental conditions had been proved long before [5, 7–14]. In vitro determination of antibiotic susceptibility of *M. synoviae* is an essential tool for the choice of the appropriate antibiotic agent in the therapy with taking in consideration the factors which may influence the antimicrobial effect in vivo (e.g. biofilm synthesis) [15]. However, the performance of the in vitro tests in the case of mycoplasmas is time-consuming and requires special techniques, thus usually it is not carried out in routine diagnostics and comparable data about the antibiotic susceptibility of *M. synoviae* strains originating from Europe are scarce in the literature also [5, 7, 13, 16, 17].

In the present study the antibiotic susceptibility profile of *M. synoviae* strains originating from Central and Eastern Europe was determined by microbroth dilution method in the case of antibiotics commonly used in veterinary practice and which have potential to be used against avian mycoplasmosis.

## Methods

*M. synoviae* strains were isolated from trachea swabs collected from turkeys and chickens originating from the Central and Eastern European region between 2014 and 2016. Production phase of the sampled chickens varied among breeders, commercial layers and broilers, while only meat-type turkeys were examined in the study (Table 1). Ethical approval and specific permission were not required for the study as all samples were collected by the authors during routine diagnostic examinations or necropsies with the consent of the owners. Trachea swabs were washed in 2 ml modified Frey’s broth medium [18] and incubated at 37 °C with 5% CO_2_ atmosphere. The broth medium consisted of 0.225 g/ml Frey Mycoplasma broth base, 20% porcine serum, 0.01% NAD, 0.01% cysteine, 200 IU/ml penicillin G, 0.5% glucose, 0.5% pyruvate and 0.005% phenol red in distilled water; all products originated from Sigma-Aldrich, Germany. Strains were gained after one-time filter cloning, minimizing the in vitro mutations of the isolates. The QIAamp DNA Mini Kit (Qiagen Inc., Hilden, Germany) was used for DNA extraction according to the manufacturers’ instructions for Gram-negative bacteria. The purity of the cultures was confirmed by a universal *Mycoplasma* PCR system targeting the 16S/23S rRNA intergenic spacer region in *Mycoplasmatales* [19] followed by sequencing on an ABI Prism 3100 automated DNA sequencer (Applied Biosystems, Foster City, CA), sequence analysis and BLAST search. Out of a total of 41 *M. synoviae* strains tested in the study 26 strains originated from Hungary, three strains each from Austria, the Czech Republic and from Ukraine, two strains from Russia and one from Serbia (Table 1). Also, three strains isolated in Slovenia between 2002 and 2008 were included in the study. The number of colour changing units (CCU) was calculated by microbroth dilution method, from the highest dilution showing colour change (red to yellow shift) after two weeks of incubation [20].

**Table 1:** Background data and initial MIC values of the isolated *Mycoplasma synoviae* strains

Strain data							MIC values (μg/ml)														
Strain ID	Host^a^	Production phase	Farm	Region	Country	Year	EFX	DIF	CTC	DX	OTC	TYL	TIL	TVN	N	SPC	LCM	LCM-SPC	TIA	VAL	FFC
MYCAV 31	ch	layer	1	Nógrád	Hungary	2014	0.625	5	0.5	0.078	1	≤0.25	≤0.25	≤0.25	32	1	1	2 (0.666/1.334)	≤0.039	≤0.039	2
MYCAV 79	t	broiler	2	Győr-Moson-Sopron	Hungary	2014	0.312	1.25	≤0.25	0.078	0.5	≤0.25	≤0.25	≤0.25	32	1	1	2 (0.666/1.334)	0.078	≤0.039	2
MYCAV 102	t	broiler	3	Vas	Hungary	2014	1.25	1.25	0.5	0.078	0.5	≤0.25	≤0.25	≤0.25	32	1	1	1 (0.333/0.667)	0.078	≤0.039	2
MYCAV 119	t	broiler	4	Komárom-Esztergom	Hungary	2014	0.312	1.25	≤0.25	0.078	≤0.25	≤0.25	≤0.25	≤0.25	32	2	1	2 (0.666/1.334)	0.078	≤0.039	2
MYCAV 130	t	broiler	5	Győr-Moson-Sopron	Hungary	2014	10	5	0.5	0.078	0.5	≤0.25	≤0.25	≤0.25	32	2	1	2 (0.666/1.334)	0.156	≤0.039	4
MYCAV 167	ch	breeder	6	Jász-Nagykun-Szolnok	Hungary	2015	0.625	0.625	0.5	≤0.039	≤0.25	≤0.25	≤0.25	≤0.25	8	1	≤0.25	0.5 (0.167/0.333)	≤0.039	≤0.039	2
MYCAV 168	ch	layer	7	Pardubice	Czech Republic	2015	>10	5	2	0.312	1	≤0.25	0.5	≤0.25	8	2	1	1 (0.333/0.667)	0.156	≤0.039	8
MYCAV 170	ch	breeder	8	Győr-Moson-Sopron	Hungary	2015	2.5	2.5	0.5	0.078	0.5	≤0.25	≤0.25	≤0.25	16	2	0.5	0.5 (0.167/0.333)	0.156	≤0.039	8
MYCAV 173	ch	layer	9	Oryol	Russia	2015	10	2.5	1	0.156	0.5	≤0.25	1	≤0.25	4	1	0.5	0.5 (0.167/0.333)	≤0.039	≤0.039	2
MYCAV 174	ch	layer	10	Oryol	Russia	2015	>10	10	4	0.312	1	≤0.25	4	≤0.25	32	2	1	2 (0.666/1.334)	0.078	≤0.039	8
MYCAV 183	ch	broiler	11	Komárom-Esztergom	Hungary	2015	1.25	1.25	0.5	0.078	≤0.25	≤0.25	≤0.25	≤0.25	>64	8	0.5	1 (0.333/0.667)	0.078	≤0.039	8
MYCAV 185	ch	breeder	12	Szabolcs-Szatmár-Bereg	Hungary	2015	>10	5	0.5	0.312	0.5	2	64	0.5	16	2	>64	2 (0.666/1.334)	0.156	≤0.039	8
MYCAV 186	ch	layer	13	Borsod-Abaúj-Zemplén	Hungary	2015	>10	2.5	2	0.156	0.5	≤0.25	≤0.25	≤0.25	64	2	0.5	0.5 (0.167/0.333)	0.312	≤0.039	2
MYCAV 188	ch	breeder	14	Zala	Hungary	2015	>10	10	4	0.312	1	≤0.25	≤0.25	≤0.25	32	2	0.5	1 (0.333/0.667)	≤0.039	≤0.039	2
MYCAV 189	ch	layer	15	Cherkasy	Ukraine	2015	10	5	8	0.312	1	≤0.25	2	≤0.25	32	2	1	1 (0.333/0.667)	0.078	≤0.039	8
MYCAV 190	ch	layer	16	Cherkasy	Ukraine	2015	>10	10	4	0.312	0.5	≤0.25	2	≤0.25	32	2	1	0.5 (0.167/0.333)	0.078	≤0.039	8
MYCAV 194^b^	ch	breeder	17	unknown	Slovenia	2002	0.625	1.25	≤0.25	≤0.039	≤0.25	≤0.25	≤0.25	≤0.25	32	4	0.5	0.5 (0.167/0.333)	0.156	≤0.039	4
MYCAV 197^c^	ch	breeder	18	unknown	Slovenia	2002	0.625	10	0.5	≤0.039	≤0.25	≤0.25	≤0.25	≤0.25	32	≤0.25	0.5	2 (0.666/1.334)	0.078	≤0.039	4
MYCAV 198^d^	ch	unknown	19	unknown	Slovenia	2008	0.625	5	≤0.25	0.156	0.5	≤0.25	≤0.25	≤0.25	16	4	0.5	1 (0.333/0.667)	0.312	≤0.039	8
MYCAV 217	t	broiler	20	Békés	Hungary	2015	2.5	10	1	0.078	≤0.25	≤0.25	0.5	≤0.25	64	1	1	1 (0.333/0.667)	0.625	≤0.039	8
MYCAV 236	ch	breeder	21	Veszprém	Hungary	2015	0.312	1.25	1	0.078	≤0.25	≤0.25	≤0.25	≤0.25	16	1	0.5	0.5 (0.167/0.333)	0.078	≤0.039	0.5
MYCAV 249	ch	layer	22	South Moravia	Czech Republic	2016	5	10	≤0.25	0.156	0.5	≤0.25	≤0.25	≤0.25	>64	4	1	1 (0.333/0.667)	0.078	≤0.039	4
MYCAV 256	ch	layer	23	South Moravia	Czech Republic	2016	10	5	1	0.156	0.5	≤0.25	≤0.25	≤0.25	>64	4	1	2 (0.666/1.334)	0.156	≤0.039	4
MYCAV 257	ch	layer	24	Fejér	Hungary	2016	>10	10	2	0.156	1	≤0.25	≤0.25	≤0.25	32	2	1	1 (0.333/0.667)	0.156	≤0.039	4
MYCAV 259	ch	breeder	25	unknown	Serbia	2016	>10	5	0.5	0.078	≤0.25	≤0.25	≤0.25	≤0.25	32	2	≤0.25	0.5 (0.167/0.333)	0.312	≤0.039	2
MYCAV 261	t	broiler	26	Komárom-Esztergom	Hungary	2016	1.25	1.25	0.5	0.078	≤0.25	≤0.25	≤0.25	≤0.25	16	2	≤0.25	0.5 (0.167/0.333)	≤0.039	≤0.039	4
MYCAV 262	t	broiler	27	Komárom-Esztergom	Hungary	2016	0.625	1.25	≤0.25	0.078	≤0.25	≤0.25	≤0.25	≤0.25	16	1	0.5	1 (0.333/0.667)	≤0.039	≤0.039	8
MYCAV 263	t	broiler	28	Győr-Moson-Sopron	Hungary	2016	1.25	1.25	0.5	0.078	≤0.25	≤0.25	≤0.25	≤0.25	8	1	≤0.25	0.5 (0.167/0.333)	≤0.039	≤0.039	2
MYCAV 268	t	broiler	29	Tolna	Hungary	2016	1.25	2.5	0.5	0.078	≤0.25	≤0.25	≤0.25	≤0.25	64	2	0.5	1 (0.333/0.667)	0.156	≤0.039	8
MYCAV 272	ch	layer	30	Ternopil	Ukraine	2016	0.625	1.25	≤0.25	0.078	≤0.25	≤0.25	≤0.25	≤0.25	>64	4	2	1 (0.333/0.667)	0.625	≤0.039	4
MYCAV 274	ch	broiler	31	Vas	Hungary	2016	2.5	2.5	≤0.25	0.078	≤0.25	≤0.25	≤0.25	≤0.25	64	4	1	2 (0.666/1.334)	0.312	≤0.039	8
MYCAV 277	t	broiler	32	Veszprém	Hungary	2016	1.25	1.25	≤0.25	0.078	≤0.25	≤0.25	≤0.25	≤0.25	32	2	0.5	1 (0.333/0.667)	0.078	≤0.039	4
MYCAV 278	t	broiler	33	Veszprém	Hungary	2016	1.25	1.25	≤0.25	0.156	≤0.25	≤0.25	≤0.25	≤0.25	16	2	0.5	1 (0.333/0.667)	≤0.039	≤0.039	2
MYCAV 281	t	broiler	34	Somogy	Hungary	2016	0.312	0.625	≤0.25	0.156	≤0.25	≤0.25	≤0.25	≤0.25	32	2	1	1 (0.333/0.667)	0.156	≤0.039	8
MYCAV 282	ch	layer	13	Borsod-Abaúj-Zemplén	Hungary	2016	10	5	0.5	0.156	0.5	≤0.25	≤0.25	≤0.25	>64	4	1	2 (0.666/1.334)	≤0.039	≤0.039	0.5
MYCAV 284	t	broiler	35	Burgenland	Austria	2016	1.25	2.5	≤0.25	0.078	≤0.25	≤0.25	≤0.25	≤0.25	32	2	0.5	1 (0.333/0.667)	0.156	≤0.039	4
MYCAV 285	t	broiler	36	Burgenland	Austria	2016	1.25	2.5	≤0.25	0.078	≤0.25	≤0.25	≤0.25	≤0.25	32	2	≤0.25	0.5 (0.167/0.333)	0.078	≤0.039	4
MYCAV 288	t	broiler	37	Burgenland	Austria	2016	1.25	2.5	≤0.25	0.078	≤0.25	≤0.25	≤0.25	≤0.25	>64	1	0.5	1 (0.333/0.667)	0.078	≤0.039	4
MYCAV 291	t	broiler	38	Győr-Moson-Sopron	Hungary	2016	0.625	2.5	0.5	0.156	0.5	≤0.25	≤0.25	≤0.25	32	1	≤0.25	0.5 (0.167/0.333)	0.078	≤0.039	4
MYCAV 300	ch	breeder	14	Zala	Hungary	2016	>10	10	1	0.312	1	≤0.25	1	≤0.25	64	2	1	1 (0.333/0.667)	0.078	≤0.039	4
MYCAV 306	t	broiler	4	Komárom-Esztergom	Hungary	2016	1.25	2.5	≤0.25	0.078	≤0.25	≤0.25	≤0.25	≤0.25	16	1	1	1 (0.333/0.667)	0.078	≤0.039	4

*Abbreviations of antibiotics*: *EFX* enrofloxacin, *DIF* difloxacin, *DX* doxycycline, *OTC* oxytetracycline, *CTC* chlortetracycline, *TYL* tylosin, *TIL* tilmicosin, *TVN* tylvalosin, *N* neomycin, *SPC* spectinomycin, *LCM* lincomycin, *TIA* tiamulin, *VAL* valnemulin, *FFC* florfenicol

^a^All strains were isolated from the trachea of the animals, abbreviations stand for: *ch* chicken, *t* turkey

^b^MYCAV194 is sub-clone of strain ULB02/T6 [41]

^c^MYCAV197 is sub-clone of strain IT2/A [41]

^d^MYCAV198 is sub-clone of strain ULB08/T3 [42]

The following antimicrobial agents were examined during the microbroth dilution tests: the fluoroquinolones: enrofloxacin and difloxacin; the aminocyclitol: spectinomycin; the aminoglycoside: neomycin; the lincosamide: lincomycin; the tetracyclines: doxycycline, oxytetracycline and chlortetracycline; the macrolides: tylosin and tilmicosin; the pleuromutilins: tiamulin and valnemulin; and the amphenicol: florfenicol; all products originated from VETRANAL, Sigma-Aldrich, Germany. Lincomycin and spectinomycin were applied in combination as well, in a ratio of 1:2. The macrolide tylvalosin (Aivlosin, ECO Animal Health Ltd., UK) was also included in the examinations. The antibiotics were diluted and stored according to the recommendations of Hannan [20]. Stock solutions of 1 mg/ml fluoroquinolones were prepared in 0.1 M NaOH; stock solution of 1 mg/ml florfenicol was prepared in 96% ethanol and in sterile distilled water; and the rest of the stock solutions of 1 mg/ml were prepared in sterile distilled water and stored at −70 °C. Freshly prepared two-fold dilutions were used in each microtest after checking the thawed antibiotic solutions for any visible changes in their consistency. The concentration range of the antibiotics was selected to cover values previously suggested to reflect susceptibility, intermediate susceptibility or resistance to the tested agents or which were used in previous publications (Table 2), in details: 0.039–10 μg/ml for fluoroquinolones, doxycycline and pleuromutilins, 0.25–64 μg/ml for neomycin, spectinomycin, lincomycin, oxytetracycline, chlortetracycline and macrolides, 0.125–32 μg/ml for florfenicol and 0.25–64 μg/ml (0.083/0.167–21.333/42.666 μg/ml) for the combination of lincomycin and spectinomycin.

**Table 2:** Summary of MIC range, MIC_50_ and MIC_90_ values (μg/ml) of the isolated *Mycoplasma synoviae* strains with the suggested non-official breakpoints (in μg/ml; S: susceptible, R: resistant) and MIC values for the type strain WVU1853

	Non-official breakpoints	WVU1853 initial	WVU1853 final	Range initial	Range final	MIC_50_ initial	MIC_50_ final	MIC_90_ initial	MIC_90_ final
Fluoroquinolones									
Enrofloxacin	S ≤ 0.5; *R* ≥ 2 [5]	0.312	1.25	0.312 – >10	0.312 – >10	1.25	1.25	>10	>10
Difloxacin	S ≤ 0.5; *R* ≥ 4 [5]	1.25–2.5	1.25–2.5	0.625 – >10	0.625 – >10	2.5	2.5	10	>10
Aminocyclitol									
Spectinomycin	S ≤ 2; *R* > 4 [28]	2	8	≤0.25–8	1–16	2	2	4	8
Aminoglycoside									
Neomycin	S ≤ 4; R > 4 [25]	64 – >64	>64	4 – >64	8 – >64	32	>64	>64	>64
Lincosamide									
Lincomycin	S ≤ 2; *R* ≥ 8 [43]	0.5	0.5	≤0.25 – >64	≤0.25 – >64	0.5	1	1	4
Tetracyclines									
Doxycycline	S ≤ 4; *R* ≥ 16 [25]	≤0.039	0.156	≤0.039–0.312	0.078–1.25	0.078	0.156	0.312	0.625
Oxytetracycline	S ≤ 4; R ≥ 16 [5]	≤0.25	0.5	≤0.25–1	≤0.25–8	≤0.25	1	1	4
Chlortetracycline	S ≤ 4; R ≥ 16 [25]	0.5	1	≤0.25–8	≤0.25–16	0.5	2	2	8
Macrolides									
Tylosin	S ≤ 1; R ≥ 4 [5]	≤0.25	≤0.25	≤0.25–2	≤0.25–8	≤0.25	≤0.25	≤0.25	≤0.25
Tilmicosin	S ≤ 8; *R* ≥ 32 [5]	≤0.25	≤0.25	≤0.25–64	≤0.25 – >64	≤0.25	≤0.25	1	2
Tylvalosin	S ≤ 0.5; *R* > 2 [28]	≤0.25	≤0.25	≤0.25–0.5	≤0.25–1	≤0.25	≤0.25	≤0.25	≤0.25
Pleuromutilins									
Tiamulin	S ≤ 8; R ≥ 16 [7]	0.078	0.078	≤0.039–0.625	0.078–1.25	0.078	0.156	0.312	0.312
Valnemulin	S ≤ 0.125; *R* > 0.125 [25]	≤0.039	≤0.039	≤0.039	≤0.039	≤0.039	≤0.039	≤0.039	≤0.039
Amphenicol									
Florfenicol	S ≤ 2; R ≥ 8 [25]	1–2	1–4	0.5–8	1–16	4	8	8	8
Combination									
Lincomycin: Spectinomycin	S ≤ 2(0.666/1.334); R > 4(1.332/2.668) [25]	1 (0.333/0.667)	1–2 (0.333/0.667–0.666/1.334)	0.5–2 (0.167/0.333–0.666/1.334)	0.5–4 (0.167/0.333–1.332/2.668)	1 (0.333/0.667)	1 (0.333/0.667)	2 (0.666/1.334)	2 (0.666/1.334)

Microbroth dilution examinations were performed according to Hannan [20] on 10^4^–10^5^ CCU/ml of the strains. In brief, the tests were performed in 96-well microtiter plates containing modified Frey’s broth medium, using growth controls (broth medium without antibiotic), sterility controls (broth medium without antibiotic and *Mycoplasma* inoculum), pH controls (broth medium adjusted to pH 6.8) and quality controls (the duplicate of the *M. synoviae* type strain WVU 1853, NCTC 10124). All strains were tested in duplicates.

The minimum inhibitory concentrations (MIC) were determined from the lowest concentration of the antibiotics where no pH and colour change of the broth was detected, meaning that the growth of the bacteria was completely inhibited in the broth. Initial MIC values were determined when the growth controls showed colour change. Final MIC values were determined when no further growth was detected, generally after two weeks of incubation. MIC_50_ and MIC_90_ values were defined as the lowest concentrations that inhibited the growth of 50% or 90% of the strains [20].

## Results

The quality control type strain (WVU 1853, NCTC 10124) showed consistent results throughout the study and the data (Table 2) were in accordance with previously recorded MIC values gained by microbroth dilution method: ranges of initial MIC values were 0.125–0.5 μg/ml for enrofloxacin and difloxacin, 0.1–0.125 μg/ml for oxytetracycline, ≤0.015 μg/ml for doxycycline, 0.025–0.06 μg/ml for tylosin, 0.015–0.06 μg/ml for tilmicosin, and ≤0.03–0.1 μg/ml for tiamulin before [5, 7, 13, 17]. Currently, there are no comparable MIC values available in the case of the *M. synoviae* type strain (WVU 1853, NCTC 10124) for the rest of the antibiotics tested in the present study. The ranges of the initial and final MIC values, MIC_50_ and MIC_90_ values for each antibiotic and for the combination are included in Table 2. In the cases of four antibiotics (oxytetracycline, chlortetracycline, neomycin and lincomycin) at least four-fold difference was observed in the MIC_50_ or MIC_90_ values when initial and final MIC values were compared (Tables 1 and 2 and Additional file 1). The initial MIC values are evaluated and discussed throughout the study [20]. The MIC_50_ values of the strains originating from different countries of the Central and Eastern European region showed high similarity, thus if otherwise not indicated the MIC values of all examined strains are evaluated together.

**Fig. 1:**
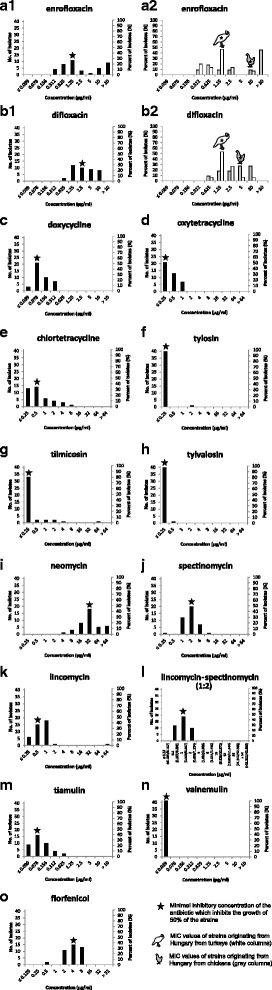
MIC50 values are marked with asterisks. MIC values of 41 *M. synoviae* strains are demonstrated to enrofloxacin (**a1**), difloxacin (**b1**), doxycycline (**c**), oxytetracycline (**d**), chlortetracycline (**e**), tylosin (**f**), tilmicosin (**g**), tylvalosin (**h**), neomycin (**i**), spectinomycin (**j**), lincomycin (**k**), the combination of lincomycin and spectinomycin (**l**), tiamulin (**m**), valnemulin (**n**) and florfenicol (**o**). The MIC values for enrofloxacin (**a2**) and difloxacin (**b2**) of isolates originating from Hungary from meat-type turkeys (white columns; n = 15) and mostly breeder and layer chickens (grey columns; n = 11) are presented in individual diagrams also

The distribution of the MIC values for enrofloxacin showed two main peaks (Fig. 1a1), while predominantly even distribution of the MIC values for difloxacin was observed (Fig. 1a2). Among the Hungarian strains the MIC_50_ values for enrofloxacin of the isolates originating from chickens (10 μg/ml, *n* = 11) was notably higher than of the strains originating from turkeys (1.25 μg/ml, *n* = 15), which corresponds to the observed two-peaked distribution (Fig. 1a2). In this comparison, four-fold difference was detected between the MIC_50_ values for difloxacin (with MIC_50_ 1.25 μg/ml and 5 μg/ml of strains from turkeys and chickens, respectively) and the distribution of the MIC values for this agent differed remarkably according to the isolates’ host of origin (Fig. 1b2). No outlier strains with high MIC values were observed for the tetracyclines doxycycline, oxytetracycline and chlortetracycline (Fig. 1c, d and e). The strains generally showed low MIC values for the three examined macrolides (Fig. 1f, g and h), with the exception of one strain (MYCAV 185), especially in the case of tilmicosin (MIC 64 μg/ml). Strain MYCAV 185 was isolated from a backyard flock, where excess antibiotic usage was documented, and it showed elevated MIC values for most antibiotics tested. High MIC values were detected for neomycin in most strains (Fig. 1i), especially after two weeks of incubation (final MIC_50_ > 64 μg/ml, Table 2 and Additional file 1). The majority of the strains’ MIC values for spectinomycin and for lincomycin distributed around the MIC_50_ values (Fig. 1j and k). Outlier strains were detected for both antibiotics; one strain with low MIC value in the case of spectinomycin (MYCAV 197), and one with high MIC value in the case of lincomycin (MYCAV 185). When lincomycin and spectinomycin were applied in combination, the range of the MIC values slightly tightened, no outlier strains were detected and lower concentration of the individual antibiotics was sufficient in the combination to inhibit the growth of 50% of the strains (Fig. 1j, k and l). Pleuromutilins showed high efficacy against the *M. synoviae* strains (Fig. 1m and n). No growth was observed in the presence of valnemulin and most strains were inhibited at the MIC_50_ concentration of tiamulin. The MIC values of the majority of the strains grouped around the MIC_50_ value (4 μg/ml) in the case of florfenicol also (Fig. 1o).

## Discussion

Conventional methods for the determination of antibiotic susceptibility of mycoplasmas is time-consuming, laborious and requires special techniques, thus it is not performed routinely [20]. Moreover, the interpretation of the results is hampered by the lack of official standards. In the case of human pathogen mycoplasmas the Clinical and Laboratory Standards Institute (CLSI) has provided official breakpoints for certain antibiotics [21]. However, given the fastidious nature and inherent differences in the cultivation of these pathogens, procedures and media vary according to each of the examined species [22]. Initiations to establish standard CLSI protocols for mycoplasmas with veterinary relevance have been made, but for the time being the recommendations of Hannan [20] are supposed to be applied in these cases [23]. In the lack of official breakpoints, the data of the present study are interpreted according to values previously used in other publications [5, 7, 13, 20, 24] or to breakpoints of other avian pathogens determined by the CLSI [25] (Table 2).

Susceptibility (MIC ≤0.5 μg/ml according to Landman et al. [5]) and also resistance (MICs ≥2 or 4 μg/ml for enrofloxacin or difloxacin, respectively) [5] to fluoroquinolones have been described in *M. synoviae* strains before [5, 12, 16, 17, 24]. Differences in the antibiotic usage and density of poultry flocks were assumed to be responsible for the observed variations [17] and resistance to fluoroquinolones was described in Europe [5]. In the present study, elevated MIC values of fluoroquinolones were observed regardless of the strains’ geographical origin. On the other hand, MIC values showed correlation with the host of origin, most probably in connection with the length of the hosts’ production cycle. In the interpretation of Landman et al. [5], more than half of the Hungarian strains isolated from chickens (mainly breeders and layers) were regarded resistant to enrofloxacin (MIC ≥2 μg/ml) and difloxacin (MIC ≥4 μg/ml), while most of the strains from meat-type turkeys (short life production cycle) were considered susceptible or intermediately susceptible to these antibiotics.

The MIC values of tetracyclines also varied in previous works according to the strains’ country of origin, with most European strains showing susceptibility (MIC ≤4 μg/ml) [20] to these agents [5, 12, 13, 16]. In accordance with previous studies, all strains included in the current study showed high susceptibility to doxycycline and oxytetracycline, and chlortetracycline proved to be highly efficient at least against 90% of the strains.

The 16-membered ring macrolides showed good in vitro activity against *M. synoviae* strains all over the world previously [5, 12, 14, 16, 26–30], but field strains showing intermediate susceptibility or resistance (MIC >1 μg/ml according to Hannan [20]) have been isolated also, even from Europe [13]. In avian *Mycoplasma* strains resistance to tilmicosin developed more readily and quicker than to tylosin under laboratory conditions [31]. Several previous studies reported the slow increase of resistance to tylosin in *M. synoviae* and *M. gallisepticum* in vitro also [7, 32, 33]. In the current examination, the majority of the strains were inhibited by low concentrations of tylvalosin and tylosin, and 90% of the strains were susceptible to tilmicosin (≤1 μg/ml) [5], confirming the high in vitro efficiency of these macrolides against *M. synoviae*. Elevated MIC values were detected primarily in the case of tilmicosin (with four strains reaching MIC >1 μg/ml), which is concordant with previous observations in vitro [31], and assumes the more rapid development of resistance against this agent.

Lincosamides have similar protein synthesis inhibitory mechanism on the 50S subunit of the bacterial ribosome as macrolides [34], and lincomycin was found to be efficacious against avian mycoplasmas before [3, 16]. Cross-resistance was described between macrolides and lincosamides and it was associated with mutations in the 23S ribosomal RNA of *M. synoviae* [35]. In the current study, all isolates showed susceptibility (≤2 μg/ml) [20] to lincomycin except for one outlier strain (MYCAV 185; MIC >64 μg/ml), which showed elevated MIC values to macrolides as well.

Aminoglycosides and aminocyclitols are most commonly administered for the treatment of bacterial enteritis in poultry [36, 37], and by the oral application these compounds absorb poorly from the gastrointestinal tract [38]. Previous in vitro examinations on the efficacy of neomycin against *M. synoviae* revealed that high concentrations of the antibiotic were needed for the inhibition of the pathogen (MICs 32–128 μg/ml) [30]. On the other hand, spectinomycin proved to be effective against the French and Iranian *M. synoviae* strains in vitro with MIC values below the susceptibility breakpoint of 4 μg/ml (according to CLSI [25]) [16, 28]. Potentially lower concentrations of spectinomycin were sufficient for the inhibition of the growth of *M. synoviae* when it was applied in combination with lincomycin [28], and this combination successfully controlled experimental *M. synoviae* infection in vivo before [9]. In the present study, the majority of the strains showed resistance to neomycin (MIC >4 μg/ml according to CLSI [25]) but were inhibited by spectinomycin at concentrations below the assumed breakpoint (MIC ≤4 μg/ml [25]). The combination of lincomycin with spectinomycin improved the efficacy of both antibiotics against most *M. synoviae* strains (Tables 1 and 2 and Additional file 1); therefore the use of their combination is supposed to be preferable in the therapy.

Pleuromutilins showed high in vitro effect against avian mycoplasmas before [7, 13] and have been used in the treatment of mycoplasmosis in poultry [39]. Resistance against these substances in *M. gallisepticum* and *M. synoviae* develops gradually [7], as only one mutation is enough for the elevation of MIC values, but to achieve high level resistance the combination of multiple mutations is required [39]. The *M. synoviae* strains examined in this study showed high susceptibility to tiamulin and valnemulin, assuming their potential in the therapy.

Phenicols are broad-spectrum antibiotics and showed in vitro activity against certain mycoplasmas before [23, 30, 38, 40]. In the present study, although two strains were inhibited by lower concentrations of florfenicol (MIC 0.5 μg/ml), narrow range of MICs was observed among the rest of the strains (MICs between 2 and 8 μg/ml), showing lower effectiveness of florfenicol against *M. synoviae* than reported in other studies or in *M. gallisepticum* [30, 40].

The observed differences between the initial and final MIC values of the mycoplasmastatic antibiotics chlortetracycline and lincomycin [34] lead to the re-categorization of certain strains from susceptible to resistant during the interpretation of the results, while in other cases no difference was detected at all. Also, remarkable deviation of the MIC values for neomycin (which has concentration-dependent mycoplasmacidal effect [34]) was observed when initial and final readings were compared, although it did not alter significantly the interpretation of the data. Many factors may influence the growth of the bacteria in the in vitro tests; the discrepancies may indicate the inactivation of the used antibiotics during incubation, or the presence of a slower growing minor population which may have significance in the determination of official breakpoints in the future and in the estimation of the in vivo efficacy of the antibiotics [20, 24, 26]. The combined examination of the in vitro tests with pharmacokinetics/pharmacodynamics studies and in vivo experiments would probably enable the better understanding of the importance of the initial and final MIC values, and the differences in between. Nevertheless, freshly prepared antibiotic solutions are administered during treatment, which minimize the possibility of antibiotic inactivation and initial MIC values are evaluated in the standardized methods for human pathogen mycoplasmas as well [22]; therefore the initial MIC values are advised to be taken into account in the interpretation of the results in mycoplasmas with veterinary relevance.

Strains originating from the same farm but from different years possessed similar MIC values (e.g. strains from farms 4, 13 and 14; Table 1). However, apart from oxytetracycline, doxycycline, tylvalosin, valnemulin and the combination of lincomycin and spectinomycin, strains with elevated MIC values were detected in the cases of all antibiotics tested. Even more, as an alarming example for irresponsible antibiotic usage, one strain (MYCAV 185) showed high MIC values to several antibiotics, especially to fluoroquinolones, macrolides and to lincomycin. It is noteworthy, that the combined application of lincomycin with spectinomycin remarkably reduced the inhibitory antibiotic concentration against this strain (from MIC_lincomycin_ > 64 μg/ml to MIC_lincomycin:spectinomycin_ 2 μg/ml (0.666/1.334 μg/ml)). All of these observations highlight the importance of testing the antibiotic susceptibility of *M. synoviae* before treatment. On the other hand, in clinical cases when rapid intervention is needed (e.g. mortality or high morbidity with severe clinical signs) and the treatment cannot wait for the results of the time-consuming and laborious in vitro tests, the presented data may serve as a guide in the choice of the appropriate antibiotic therapy in the Central and Eastern European region.

## Conclusions

Antibiotic susceptibility testing of *M. synoviae* is laborious and time-consuming, and is not performed in routine diagnostics, thus empirical antibiotic treatment is usually applied by the clinicians. The MIC values of the 41 *M. synoviae* strains provided in the present study revealed the in vitro effectiveness of tetracyclines, macrolides and pleuromutilins, and assume the potential usefulness of these agents in the therapy of mycoplasmosis in poultry in Central and Eastern Europe. However, elevated MIC values were observed in several cases during the examinations, which concerns antibiotics with importance in human medicine as well (e.g. fluoroquinolones). In order to preserve these critical antimicrobials for the therapy of humans, prudent antibiotic usage is recommended based on preliminary in vitro antibiotic susceptibility tests and on the careful evaluation of these data by considering the difficulties in the interpretation of the results and the factors influencing antibiotic effectiveness in vivo.

## Additional file

Additional file 1: Background data and initial and final MIC values of the isolated *Mycoplasma synoviae* strains. (XLS 61 kb)

## Abbreviation

MIC: Minimal inhibitory concentrations
